# Synthesis of Phosphorus(V)-Substituted Six-Membered *N*-Heterocycles: Recent Progress and Challenges

**DOI:** 10.3390/molecules28062472

**Published:** 2023-03-08

**Authors:** Yulia Volkova, Igor Zavarzin

**Affiliations:** N. D. Zelinsky Institute of Organic Chemistry, Russian Academy of Sciences, 47 Leninsky Prosp., 119991 Moscow, Russia

**Keywords:** heterocycles, pyridine, pyridazine, pyrimidine, pyrazine, organophosphorus compounds

## Abstract

Heterocycles functionalized with pentavalent phosphorus are of great importance since they include a great variety of biologically active compounds and pharmaceuticals, advanced materials, and valuable reactive intermediates for organic synthesis. Significant progress in synthesis of P(O)R_2_-substituted six-membered heterocycles has been made in the past decade. This review covers the synthetic strategies towards aromatic monocyclic six-membered N-heterocycles, such as pyridines, pyridazines, pyrimidines, and pyrazines bearing phosphonates and phosphine oxides, which were reported from 2012 to 2022.

## 1. Introduction

Pentavalent phosphorus-substituted heterocycles are organophosphorus compounds of great importance. In materials chemistry, they are used in design of dyes and polymers with outstanding characteristics [1]. They are of interest as Lewis acids and ligands in metal-catalyzed organic reactions [2,3,4,5]. Moreover, phosphorus(V)-substituted heterocycles exhibit a wide range of biological activities. In particular, pyridines were used to design anticancer [6,7,8], antidiabetic [9], and lusitropic agents [10], antioxidants [11], human glucokinase activators [12], NMDA receptor antagonists [13], and metalloprotein ligands [14]. Phosphorus-modified pyridazines are promising candidates as antimicrobial agents [15] and foliar herbicides [16]. Phosphorus-containing pyrimidines are used as inhibitors of dihydroorotate dehydrogenase and hepatitis C virus polymerase [17,18,19]. Pyrazines are of interest as modulators of the human glucagon-like peptide-1 receptor [20] and cyclin-dependent kinases [21].

A variety of methodologies for synthesis of pentavalent phosphorus-substituted heterocycles and their derivatives have been reported in past years. Meanwhile, development of novel methodologies is in continuous demand. Of particular interest are methods that provide a broad substrate scope and provide products with high atom economy, molecular complexity, and great efficiency under mild conditions. Among them, several eco-friendly approaches have been developed by us [22,23]. In general, the known methods for synthesis of phosphorus(V)-substituted aromatic heterocycle are classified into two synthetic strategies: (1) construction of a heterocyclic core using phosphorus-containing reagents and (2) functionalization of a pre-synthesized heterocyclic core with phosphorus reagents. 

Several excellent reviews dealing with synthesis of phosphorus-containing heterocyclic compounds were published in the literature in recent years. A number of reviews highlighted phosphorylation as the most common approach [24,25] and specifically radical phosphorylation [26] and metal-catalyzed/promoted C–H bond phosphorylation [27]. Another array of reviews considered use of a specific kind of transformations, including photoredox-catalyzed reactions [28], electrocatalytic [29], and radical [30] C–P bond formation, multicomponent reactions [31], and functionalization of phosphorus-centered radicals [32]. Some reviews are focused on the type of P-functional groups [33] or type of heterocycles [34], e.g., phosphorylindoles [35,36], pyrazoles [37,38], or pyrrolidines [39]. However, systematic reviews of the published data on different methods for synthesis of most monocyclic phosphorus(V)-substituted six-membered *N*-heterocycles are lacking.

The main focus of this review is monocyclic six-membered aromatic P(O)R_2_-substituted *N*-heterocycles, including pyridines, pyridazines, pyrimidines, and pyrazines. The review is an attempt to systematically consider and classify methods for synthesis of corresponding heterocyclic phosphonates and phosphine oxides. Phosphonic acids are excluded from consideration since most of them are available by saponification of phosphonates [40,41,42,43,44,45]. Synthesis of six-membered *N*-heterocyclic phosphinamides, phosphonamide, and phosphonamidate has also not been described in recent years. The review is organized in a classical way and includes classification according to type of *N*-heterocycles using the following criteria: (a) the number of nitrogen atoms in heterocycles, (b) the nature of synthetic precursors, and (c) the type of chemical transformations involving these heterocycles. 

The review addresses the scientific advances made over the past decade, in the period from 2012 up to the end of 2022. However, some earlier pioneering studies are cited where necessary. In many cases, the reaction mechanisms are briefly discussed in order to more completely describe the synthetic approaches to phosphorus-substituted *N*-heterocycles. In order to avoid overloading of the text with names of semantically similar processes, the term “phosphorylation” in this review deals with introduction of any P(O)R_2_ substituent, not only a phosphoryl moiety, into the heterocyclic core. To enhance the readability of the schemes, the following color coding of the reagents is used: the source of the nitrogen atom is shown in blue, the source of the phosphorus atom in red, and the extra components in green. In the case of intramolecular cyclizations of reagents containing both nitrogen and phosphorus, the nitrogen atom is marked in blue and the phosphorus moiety in red.

## 2. Pyridines

**Figure 1:**
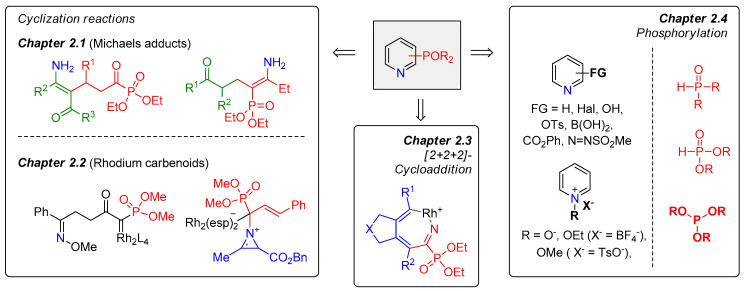
Approaches to synthesis of POR_2_-containing pyridines.

Phosphorus(V)-substituted pyridine was first synthesized by Plazek’s research group [46] in 1936 by a reaction of 2-dimethylaminopyridine with phosphorus trichloride under oxidative conditions. Later, related compounds were obtained by reactions of metalated pyridines with phosphorus-halogen compounds [47,48,49], of pyridinediazonium tetrafluoroborate with phosphorus trichloride [50], of *N*-alkoxypyridines with sodium diethyl phosphite and phosphines [51,52,53,54,55], and of *N*-pyridylpyridines with phosphonic acid [56], by the Michaelis–Arbuzov reaction [57,58], Pd(II)-catalyzed phosphorylation of halopyridines [45,59,60], cyclization of phosphorus-containing 3-azatrienes [61], and the Diels–Alder reaction involving 3-phosphoryl-1-azadienes [62]. This section of the review considers recent examples of synthesis of POR_2_-substituted pyridines, including the intramolecular cyclizations of Michael adducts, insertion of carbenoids, formal [2+2+2]-cycloaddition, and phosphorylation reactions (Figure 1).

### 2.1. Cyclizations Based on the Michael Reaction

**Scheme 1:**
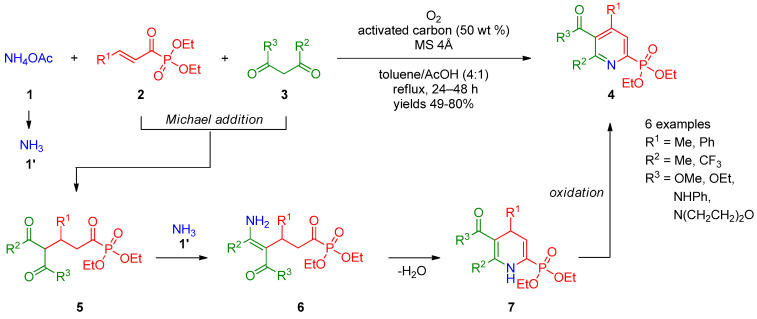
Synthesis of pyridine-2-phosphonates.

In recent years, synthesis of pyridines via the Michael reaction using phosphoryl-substituted Michael acceptors and donors has gained significant attention. Allais et al. [63,64,65] described three-component condensation of 1,3-dicarbonyl compounds **3** with P(O)Et_2_-bearing vinyl ketones **2** and ammonium acetate (**1**) in the presence of oxygen, leading to pyridine-2-phosphonates **4** (Scheme 1). The authors suggested that the reaction occurs through successive addition of compound **3** to Michael acceptors **2, providing** ketone **5** followed by enamination of the latter with ammonia (**1′**) to form intermediate **6**, which undergoes intramolecular cyclization into dihydropyridine **7**. Complete oxidation of intermediate **7** to pyridine **4** was achieved using oxygen in the presence of activated carbon. The reaction was general with respect to β-oxo esters and β-oxoamides, providing products in 49–80% yields.

**Scheme 2:**
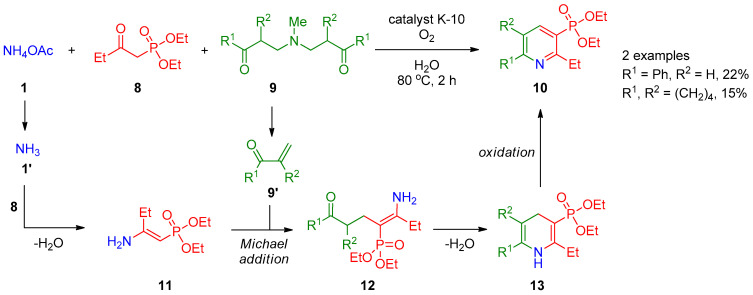
Reaction of diethyl (2-oxobutyl)phosphonate with Mannich bases.

Hanashalshahaby and Unaleroglue [66] showed that pyridine-3-phosphonates **10** can be obtained by three-component oxidative coupling of diethyl (2-oxobutyl)phosphonate (**8**) with Mannich bases **9** and ammonium acetate (**1**) in the presence of catalyst K-10 (Scheme 2). The product yields are reasonable both for aryl- and alkyl-substituted Mannich bases. The authors hypothesized that β-keto phosphonate **8** reacts with ammonia **1′** generated in situ from ammonium acetate (**1**) to form enamine **11**, which is accompanied by thermal decomposition of Mannich base **9,** giving α,β-unsaturated carbonyl compound **9′**. These two intermediates are subjected to the Michael addition to form ketoamine **12**, which undergoes intramolecular cyclization to dihydropyridine **13,** followed by oxidation of the latter with atmospheric oxygen to provide final product **10**. 

Further, Abdou et al. [67] found that diethyl (2-amino-2-thioxoethyl)phosphonate acts as an efficient Michael donor in the addition reaction with β-(dimethylamino)vinyl ketone, thus leading to 2-thioxopyridin-3-ylphosphonate. Liao et al. [68] described one example of the aza-Michael reaction between diethyl (3-phenyl-3-oxopropyn-1-yl)phosphonate with methyl 3-aminocrotonate, providing phosphonate-ester-containing pyridine moiety under mild conditions.

### 2.2. Carbenoid-Mediated Reactions

**Scheme 3:**
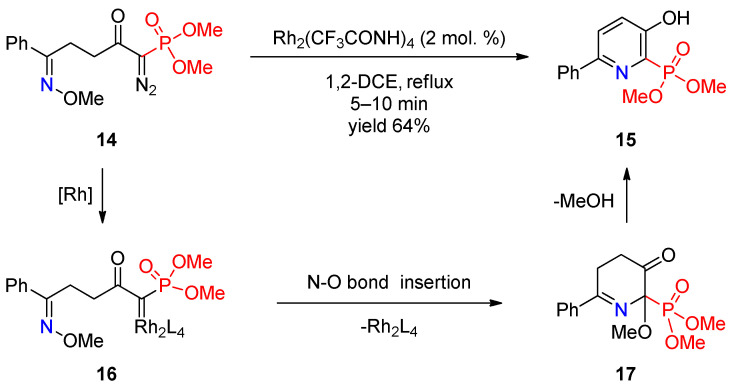
Rh(II)-catalyzed cyclization of δ-diazo oxime ether.

Recently, several studies by Park and co-workers demonstrated the prospects of using metal carbenoids in synthesis of phosphoryl-substituted pyridines. They described [69] Rh(II)-catalyzed intramolecular cyclization of δ-diazo oximes to pyridines and showed that this is a facile method for synthesis of pyridine-2-phosphonate **15** (Scheme 3). Rh_2_(CF_3_CONH)_4_ was used as the catalyst of choice. The authors proposed a mechanism that involves reaction of the diazo group of oxime **14** with Rh(II), providing rhodium carbenoid **16,** followed by insertion of the latter into the N–O bond to form dihydropyridinone **17**, which undergoes aromatization to final pyridine **15** via elimination of methanol. 

**Scheme 4:**
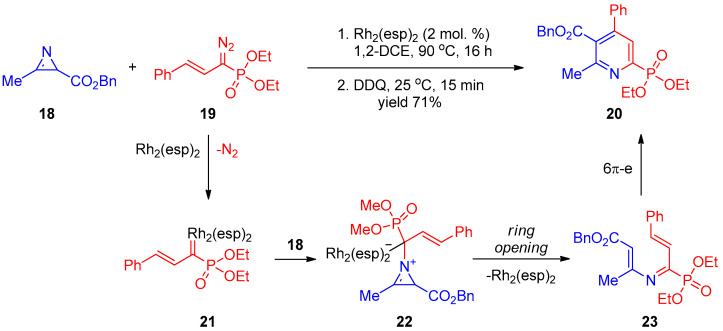
Reaction of phosphorylated carbenoid with *2H*-azirine.

In another work by Park and co-workers [70], synthesis of pyridine-2-phosphonate **20** was accomplished using phosphorylated vinyl carbenoid **21** generated in situ from diazophosphonate **19** and dirhodium(II) catalyst Rh_2_(esp)_2_ (Scheme 4). Addition of compound **21** to *2H*-azirine **18** affords intermediate **22**. The latter undergoes three-membered ring opening accompanied by elimination of the Rh(II) catalyst to provide 3-azahexatriene **23,** followed by cyclization to yield pyridine **20**. 

### 2.3. Formal [2+2+2]-Cycloaddition

**Scheme 5:**
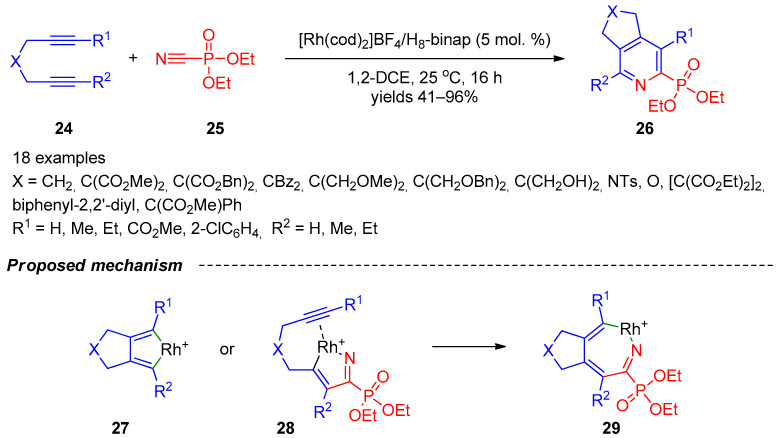
Formal [2+2+2]-cycloaddition of 1,6- and 1,7-diynes with diethyl phosphorocyanidate.

An approach towards phosphoryl-substituted pyridines, which is probably one of the most versatile, is based on the formal [2+2+2]-cycloaddition. Tanaka’s research group [71,72] described synthesis of annulated pyridine-2-phosphonates **26** based on rhodium(I)/biaryl-bisphosphine-complex-catalyzed cycloaddition of 1,6- and 1,7-diynes **24** with diethyl phosphorocyanidate (**25**) (Scheme 5). The reaction has a broad scope with respect to diynes since quaternary carbon-, methylene-, nitrogen-, and oxygen-linked internal 1,6-diynes and terminal biaryl-linked 1,7-diynes can be involved in the heterocyclization. Steric and electronic variations in diynes had minimal impact on the efficacy of the reaction, but, in some cases, using unsymmetrically substituted diynes, the reactions afforded mixtures of regioisomers. The authors proposed rhodium cyclopentadiene **27** or rhodium azacyclopentadiene **28** as two possible key intermediates in the reaction (Scheme 5). Ring expansion of both cyclopentadienes can provide seven-membered intermediate **29**, and reductive elimination of Rh(I)^+^ from the latter accomplishes formation of the final product **26**. Based on the outcome of the enantioselective version of the reaction, the authors were inclined to believe that the main reaction pathway involves intermediate **28**.

### 2.4. Phosphorylation of Pyridines

Phosphorylation is definitely the most general route to phosphorus-substituted pyridines. Due to a wide range of available phosphorylating agents and the possibility of performing the reaction in a nucleophilic, electrophilic, or radical manner, phosphorylation has attracted great attention. Such methods as the Arbuzov reaction, Hirao coupling, palladium-catalyzed cross-coupling of diethyl phosphonate with halogen-substituted heterocycles, and C(sp2)H-phosphorylation of heterocycles with diethyl phosphonates promoted by one-electron oxidants were extensively developed in past decades. Their specific applications in recent years are discussed in more detail later in the text.

#### 2.4.1. Radical Phosphorylation of Pyridines

**Scheme 6:**
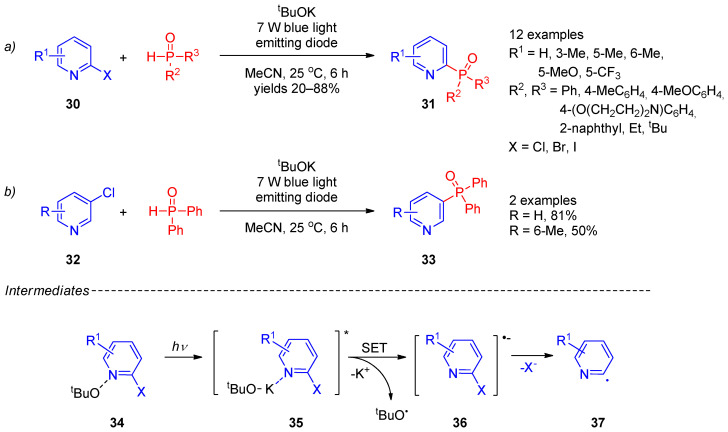
Photocatalytic phosphorylation of halopyridines: (**a**) Synthesis of 2-phosphine oxide-substituted pyridines; (**b**) Synthesis of 3-phosphine oxide-substituted pyridines.

P-centered radicals can easily be generated through hydrogen atom transfer or single-electron transfer using peroxides, metal salts, and photocatalysts. Thus, radical phosphorylation has become an efficient strategy for synthesis of structurally diverse phosphorus(V)-substituted pyridines. Due to environmental friendliness and potential industrial application, photocatalytic radical phosphorylation of the pyridine ring received extra attention. In 2018, Yuan et al. [73] reported synthesis of 2- and 3-phosphine oxide-substituted pyridines **31** and **33** (Scheme 6, lines a,b) from 2- and 3-halopyridines **30** and **32** by photocatalytic functionalization with secondary phosphine oxides in the presence of ^t^BuOK. The reaction occurred under mild conditions using irradiation with a blue-light-emitting diode. The scope of this transformation is somewhat limited to pyridines with electron-donating substituents and their analogs with an extended π-system. The plausible reaction mechanism involves formation of a complex of halopyridine with potassium *tert*-butoxide **34**, absorption of a light quantum, transition to an excited state **35**, and electron transfer from *tert*-butoxide to a pyridine ring to form the *tert*-butoxy radical and halopyridine radical anion **37**. Elimination of halide from radical anion **36** affords aryl radical **37**. Simultaneously, the *tert*-butoxy radical causes the proton abstraction from secondary phosphine to form a phosphorus-containing radical. Recombination of the latter radical with an aryl radical affords the final product. Recently, the approach proposed by Yuan et al. was expanded [74,75], including electron-primed photoredox [76] and visible light-induced nickel-catalyzed photoredox [77] conditions for radical generation.

**Scheme 7:**
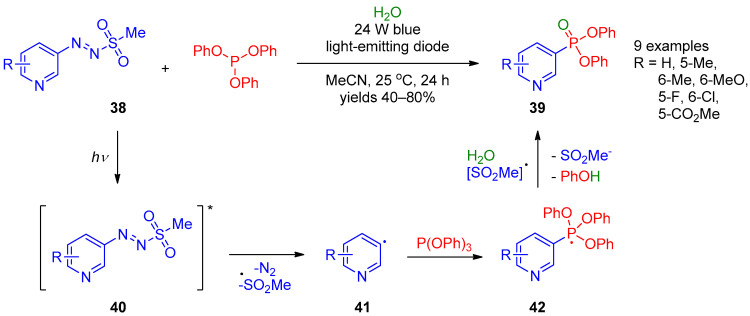
Synthesis of pyridine-3- and pyridine-4-phosphonates from pyridylazo sulfones.

In 2019, the photocatalytic radical reaction of pyridylazo sulfones **38** with triphenyl phosphite giving pyridine-3-phosphonates **39** was described by Qiu et al. [78] (Scheme 7). The reaction occurred in the presence of water. A variety of substituted pyridines can be efficiently employed in this reaction. The proposed mechanism for this transformation involves excitation of arylazo sulfone **38** under visible light to provide radical **40**, its decomposition into sulfonyl and aryl radicals, along with extrusion of a nitrogen molecule. Then, aryl radical **41** reacts with triphenyl phosphate to form phosphorus-centered radical **42,** followed by its oxidation with the sulfonyl radical and elimination of phenol to provide the final product **39**.

**Scheme 8:**
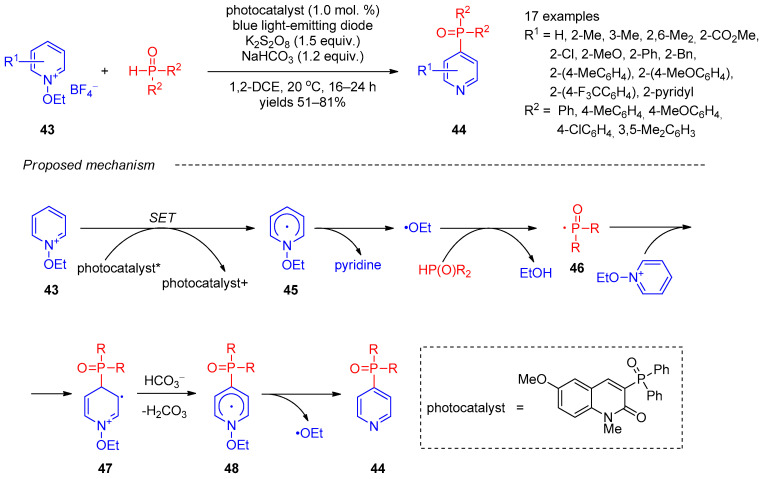
Photocatalytic reaction of *N*-ethoxypyridinium salts with phosphine oxides.

Independently, in 2019, Kim et al. [79] described site-selective synthesis of pyridines **44** bearing phosphine oxide moieties at C-4 using radical coupling of *N*-ethoxypyridinium salts **43** with secondary phosphine oxides under photocatalytic conditions (Scheme 8). Further, 3-Diphenylphosphoryl-6-methoxy-1-methyl-2(1*H*)-quinolinone under blue-light-emitting diode illumination was used as a photocatalyst and potassium persulfate as an oxidant. Examination of the reaction scope revealed that a variety of electron-withdrawing and electron-donating substituted pyridines, as well various aryl-substituted phosphine oxides, were tolerated. The plausible mechanism of this reaction involves single-electron transfer from the photocatalyst to *N*-ethoxypyridinium **43,** giving radical **45**, which undergoes decomposition accompanied by elimination of pyridine. The remaining ethoxy radical abstracts a proton from phosphine, followed by addition of the resulting phosphinyl radical **46** to *N*-ethoxypyridinium **43**. The subsequent deprotonation of intermediate **47** and elimination of the new ethoxy radical from intermediate **48** afford target product **44**. The origin of reaction chemoselectivity was revealed by DFT calculations, showing that phosphinoyl radicals are too large for providing an electrostatic attraction between the its oxo functionality and the pyridine nitrogen crucial for *ortho* functionalization. 

**Scheme 9:**
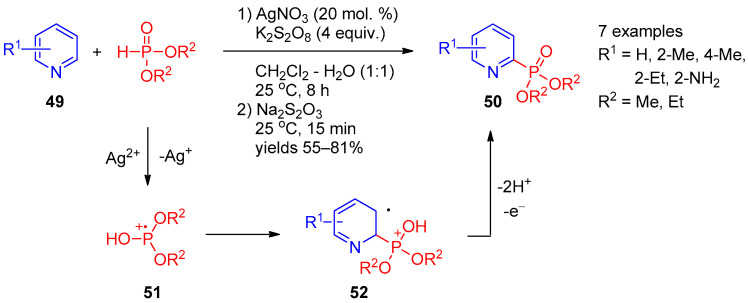
Oxidative coupling of pyridines with dialkyl phosphites in the presence of Ag(I).

Apart from the approaches based on photocatalytic radical phosphorylation, several examples using metal salts for P-radical generation were described. Huang and co-workers [80] developed a CH-phosphorylation method for synthesis of pyridinyl-2-phosphonates **50** based on Ag(I)-catalyzed reaction of pyridines **49** with dialkyl phosphonates using potassium persulfate as an oxidant (Scheme 9). An interesting feature of this reaction is that it involves subsequent treatment of the reaction mixture with sodium thiosulfate, which makes it possible to significantly increase the product yield due to a reduction of pyridine *N*-oxide formed as a by-product. In the authors’ opinion, this transformation proceeds through a radical pathway and begins with oxidation of Ag(I) to Ag(II) with persulfate, followed by oxidation of dialkyl phosphite with Ag(II) to form radical cation **5**. Addition of the latter at 2 position of the pyridine ring provides intermediate **52**. Subsequent abstraction of two protons accompanied by oxidation of radical **52** affords the final product. Recently, Kittikool et al. [81] expanded the scope of this phosphorylation approach to 2-pyridones using Mn(OAc)_2_ as the catalyst. 

Noteworthy also is a one-pot three-step protocol for synthesis of diphenyl(pyridin-2-yl)phosphine oxide based on the KOH-promoted oxidative radical phosphorylation of 2-bromopyridine with diphenylphosphine developed by Chen et al. [82].

#### 2.4.2. Nucleophilic Phosphorylation of Pyridines

**Scheme 10:**
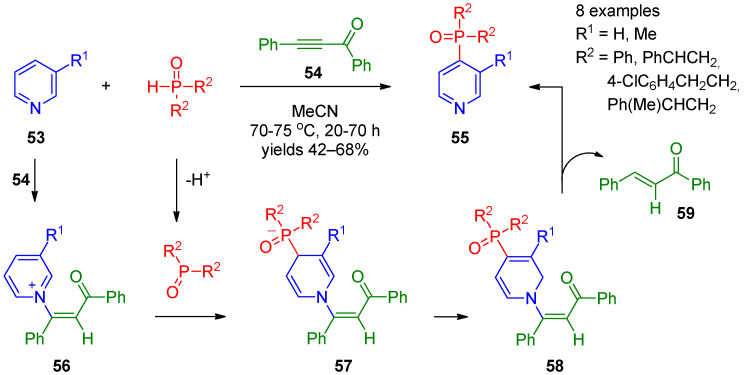
Coupling of pyridines with secondary phosphine oxides in the presence of acetylenes.

Studies by Trofimov’s research group [83,84,85] have contributed to recent progress in nucleophilic phosphorylation of pyridines at the 4 position. They accomplished synthesis of 4-phosphine oxide-substituted pyridines **55** by coupling of pyridines **53** with secondary phosphine oxides using diphenyl ethynyl ketone (**54**) as the oxidant (Scheme 10). A variety of substituted aryl and alkyl phosphine oxides can efficiently be employed in this reaction. The proposed mechanism for this transformation involves aza-Michael reaction of pyridine **53** with acetylene **54,** providing intermediate **56,** and deprotonation of phosphine, resulting in formation of the anion. Subsequent addition of a phosphine anion at the 4 position of the activated pyridine ring affords dihydropyridine **57**. The latter undergoes isomerization to intermediate **58,** followed by elimination of alkene **59** to form the final product. In this approach, 3-phenyl-2-propynenitrile can also be applied as an oxidant [84], The reaction can be stopped at the of step of 1,4-dihydropyridines **57** using terminal acylacetylenes [85]. It is interesting that 1,4-dihydropyridines are shown to be produced through 2-4 migration of the POR_2_ group in 1,2-dihydro adducts during vinylation/phosphorylation of pyridines [85]. DFT calculations supported the hypotheses that 1,4-dihydropyridines are the thermodynamic products while their 1,2-regioisomers are the kinetic ones [85].

**Scheme 11:**
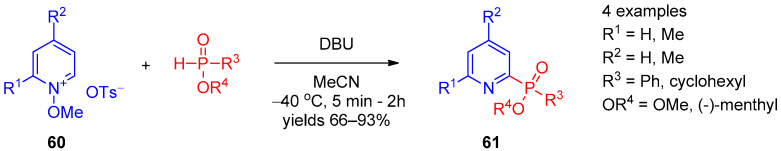
Reaction between *N*-methoxypyridinium tosylates and secondary phosphines.

Direct nucleophilic phosphorylation at the 2 position of the pyridine ring was described in 2012 by Oka et al. [86] A series of 2-phosphinate-substituted pyridines **61** were prepared from *N*-methoxypyridinium tosylates **60** by reaction with secondary phosphinates (Scheme 11). Presumably, the reaction proceeds via the S*_N_*Ar mechanism. Further, 1,8-Diazabicyclo[5.4.0]undec-7-ene (DBU) in acetonitrile at low temperature was found to be the optimal base for chemoselective transformation. The procedure was general for thiophosphinate, providing 2-pyridyl thiophosphinate. The 2-pyridyl phosphinate derivatives produced by this method are P-chiral, and the reaction can be accomplished with high diastereoselectivity using chiral phosphines.

**Scheme 12:**
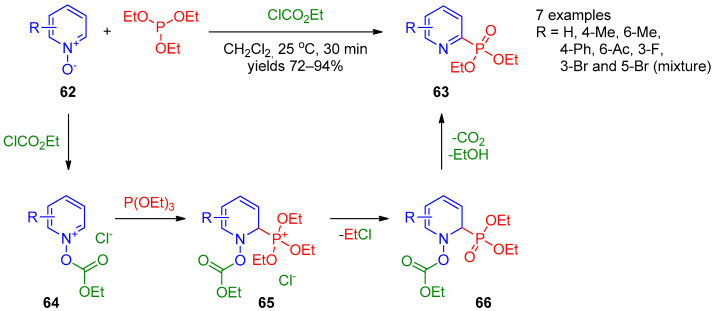
Reaction of pyridine *N*-oxides with triethyl phosphite.

In 2014, Wang et al. [87] showed that synthesis of pyridine-2-phosphonates can be accomplished directly from pyridine *N*-oxides and dimethyl phosphate over long-term heating in toluene. Independently, Lee et al. [88] developed an approach to synthesize diethyl pyridine-2-phosphonates **63** by reaction of pyridine *N*-oxides **62** with triethyl phosphite in the presence of ethyl chloroformate (Scheme 12). This reaction presumably starts with activation of pyridine *N*-oxide **62** by chloroformate, followed by addition of triethyl phosphate to the resulting pyridinium salt **64** to form intermediate **65**. The nucleophilic attack of the chloride anion at the ethyl moiety followed by elimination of ethyl carbonate from 1,2-dihydropyridine **66** affords the final product. This approach is general for preparation of quinolin-2-ylphosphonates. An apparent limitation of this approach is formation of phosphorylated isomeric mixtures in the case of 3-bromopyridine *N*-oxide.

**Scheme 13:**
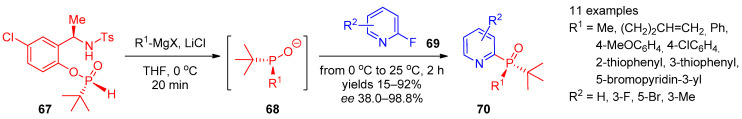
Asymmetric synthesis of tertiary pyridine-containing phosphine oxides.

Recently, Tsantrizos and co-workers [89] proposed a route to P-chiral 2-phosphine oxide-substituted pyridines (Scheme 13). Using the reaction of (*R*)-*N*-(1-(5-chloro-2-hydroxyphenyl)ethyl)-4-methylbenzenesulfonamide (**67**) with Grignard to generate chiral phosphorylating agent **68**, they accomplished enantioselective synthesis of products **70** from 2-fluoropyridines **69**. 

#### 2.4.3. Transition-Metal-Catalyzed Phosphorylation of Pyridines

Transition metals are widely used to increase efficiency of conventional non-catalyzed phosphorylation of heterocycles [27]. In the past decade, significant progress in the field of phosphorylation of pyridines was made due to application of palladium and nickel catalysis, which enabled expansion of the range of substrates active in phosphorylation reactions to involve halogen-, hydroxyl-, boronic acid-, triflate-, nonaflate-, ester-substituted, trimethylammonium pyridines, etc. It was also shown that transition-metal-catalyzed phosphorylation with phoshine oxides, phosphates, and phosphites could be efficiently used for construction of functionalized pyridines, including chiral structures. Meanwhile, transition-metal-catalyzed C–H phosphorylation of pyridines still remains an unsolved challenge.

**Scheme 14:**
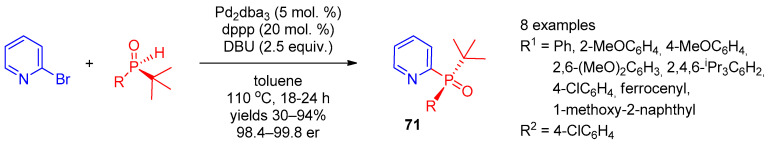
Synthesis of chiral tertiary pyridine-containing phosphine oxides.

Halopyridines (Hal = Cl, Br, and I) are efficient precursors for synthesis of 2,3,4-POR_2_-substituted pyridines under palladium catalysis conditions. In 2017, Han et al. [90] reported stereoselective palladium-catalyzed cross-coupling of 2-bromopyridine with chiral *tert*-butyl-containing phosphine oxides (Scheme 14). Use of Pd_2_(dba)_3_ with the dppp ligand enabled synthesis of tertiary pyridine-containing phosphine oxides **71** with excellent selectivity. In 2016, Dziuganowska et al. [13] used the same catalytic system to prepare a series of phosphonopyridinecarboxylic acid esters from appropriate bromides. 

**Scheme 15:**
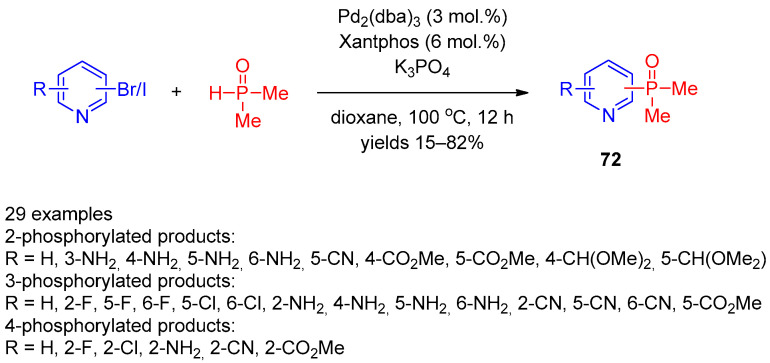
Cross-coupling of halopyridines with dimethylphosphine oxide.

Mykhailiuk and co-workers [91] expanded the scope of this reaction using the Pd_2_(dba)_3_/Xantphos catalytic system in the reaction of bromo(iodo)pyridines with dimethylphosphine oxide (Scheme 15). The scope of this reaction was found to be quite general and enables facile preparation of 2-, 3-, and 4-dimethylphosphine-oxide-substituted pyridines **72** in up to good yields. A variety of electron-withdrawing and electron-donating aryl-substituted pyridines successfully participated in the reaction. 

Borisova and co-workers [92] accomplished synthesis of bis(phosphoryl)pyridines and 2,2′-bipyridines using palladium acetate/dppf-catalyzed cross-coupling of chloropyridines with secondary phosphine oxides. Ligand-free microwave-assisted Pd(OAc)_2_-catalyzed phosphorylation of bromopyridines with diphenylphosphine oxide or diethyl phosphite was described by Henyecz et al. [93]. Catalyst Pd(OAc)_2_ in the presence of sodium iodide as the promoter was also shown to be an effective catalyst for ligand-free coupling of diphenylphosphine oxide with pyridinium nonaflate [94]. An example of Pd(PPh_3_)_4_-catalyzed coupling of 2-bromo-6-pyrrolylpyridine with diethyl phosphite was recently reported by Ti et al. [95].

**Scheme 16:**
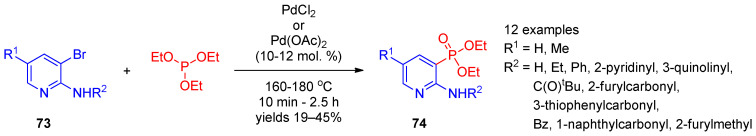
Pd(II)-catalyzed cross-coupling of 3-bromopyridines with triethyl phosphite.

The palladium-catalyzed Hirao coupling of bromopyridines with triethyl phosphite enables preparation of pyridinephosphonates. Adam et al. [96] used the palladium-catalyzed cross-coupling of 3-bromopyridines **73** with triethyl phosphite to synthesize 2-aminopyridine-3-phosphonates **74** (Scheme 16). It is worth noting that the reaction was performed in the absence of the solvent during short-term heating to 160–180 °C. 

**Scheme 17:**
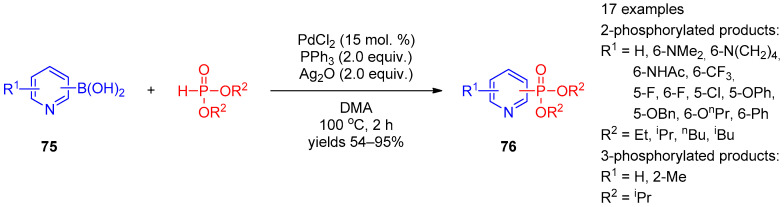
Cross-coupling of pyridine carboxylic acids with dialkyl phosphites.

Apart from halopyridines, pyridine boronic acids and hydroxypyridines were involved in phosphorylation under palladium catalysis. Zou, Wu, and co-workers [97] constructed a C–P bond by means of cross-coupling of pyridine boronic acids **75** with dialkyl phosphonates under PdCl_2_ catalysis conditions (Scheme 17). The reaction requires the presence of Ag_2_O as the oxidant. The method can be used to prepare structurally diverse pyridine-3- and pyridine-4-phosphonates **76**.

**Scheme 18:**
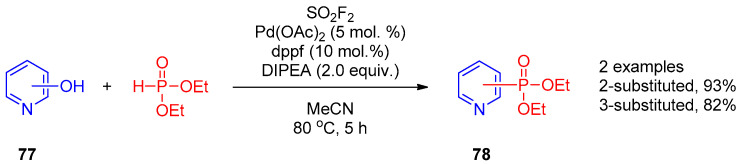
Replacement of the OH group of hydroxypyridines mediated by sulfuryl fluoride.

Recently, Ding and co-workers [98] described the general palladium-catalyzed one-pot procedure for synthesis of phosphonates, phosphinates, and phosphine oxides from phenols mediated by sulfuryl fluoride. The reaction was efficient for functionalization of 2- and 3-hydroxypyridines **77** with diethyl phosphonate (Scheme 18). According to the proposed mechanism, the fluorosulfates generated in situ are key intermediates in synthesis of pyridines **78**.

**Scheme 19:**
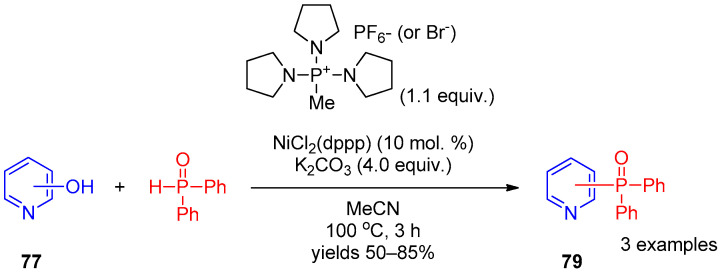
Replacement of the OH group of hydroxypyridines by the diphenylphosphoryl moiety.

Before these works, in 2012, direct replacement of an OH group of hydroxypyridines by a diphenylphosphoryl moiety was achieved by Zhao et al. [99] under Ni(II) catalysis. A C–O bond was activated using bromotripyrrolidinophosphonium hexafluorophosphate. The activated complex of hydroxypyridine **77** with this salt reacts with diphenylphosphine oxide in the presence of dichloro[1,3-bis(diphenylphosphino)propane]nickel salts as the catalyst to produce pyridines **79** (Scheme 19). 

Later, the substrate scope of Ni-catalyzed phosphorylation of pyridines with phosphine oxides was expanded to pyridine carboxylic acid phenyl esters [100] and trimethylammonium triflate of pyridine [101]. Cross-coupling of pyridine tosylates [102] and aryltrimethylammonium tetrafluoroborate [103] with phosphites was also implemented. 

**Scheme 20:**
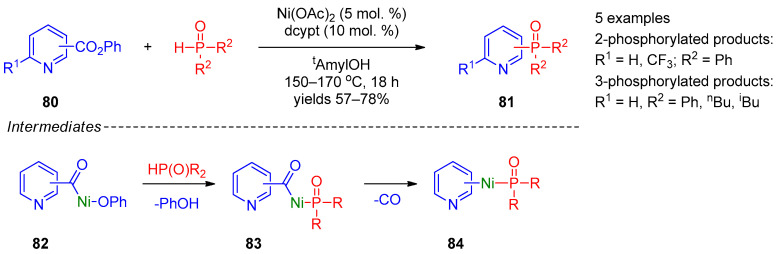
Cross-coupling of aryl carboxylic acid esters with phosphine oxides.

A relatively general method was proposed by Yamaguchi’s research group [100]. They performed cross-coupling of pyridine carboxylic acid phenyl esters **80** with secondary phosphine oxides accompanied by decarbonylation (Scheme 20). The reaction is catalyzed by nickel acetate and requires high temperatures (150–170 °C). This approach is applicable to synthesis of 2- and 3-phosphine-oxide-substituted pyridines **81**, the position of the POR_2_ substituent in the product being defined by the position of the carbonyl group in the starting compound. The authors suggested that this transformation initially proceeds through oxidative insertion of Ni(0) into the C–O bond, providing intermediate **82**, and exchange of the phenoxide ligand by phosphine provides **83**. Subsequent decarbonylation of intermediate **83** and reductive elimination from intermediate **84** afford the final product.

**Scheme 21:**
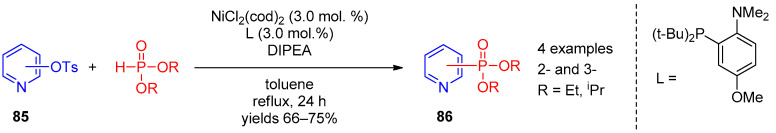
Ni(II)-catalyzed replacement of the tosyl group in pyridines.

Of interest as well is Ni(II)-catalyzed reaction of tosylates **85** with H-phosphonate diesters proposed by Chun-Jing Li [102] as an approach to 2- and 3-phosphorylpyridines **86**; NiCl_2_(cod)_2_ was an optimal catalyst for this transformation (Scheme 21).

## 3. Pyridazines

**Figure 2:**
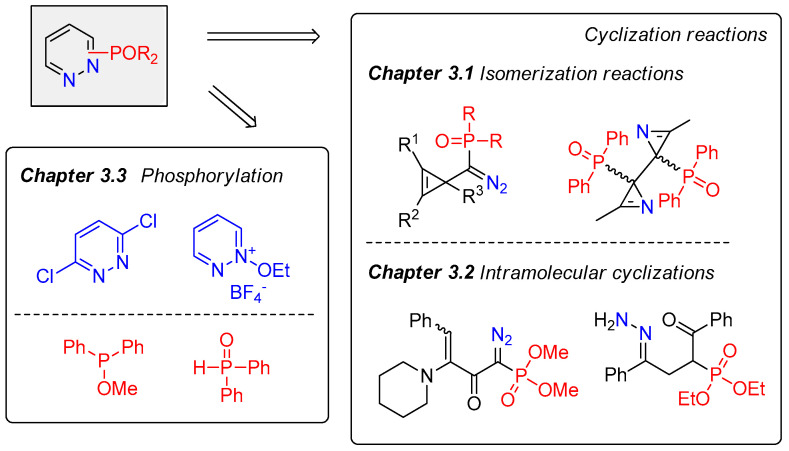
Approaches to synthesis of POR_2_-containing pyridazines.

Phosphorus(V)-substituted pyridazines are poorly described heterocycles. Therefore, all known methods for synthesis of these compounds, including isomerization of (diazomethyl)cyclopropenes and bis-azirine, intramolecular cyclization of γ,δ-unsaturated α-diazo-β-ketone and hydrazone, as well as phosphorylation of the pyridazine ring (Figure 2), are considered below. 

### 3.1. Isomerization Reactions

**Scheme 22:**
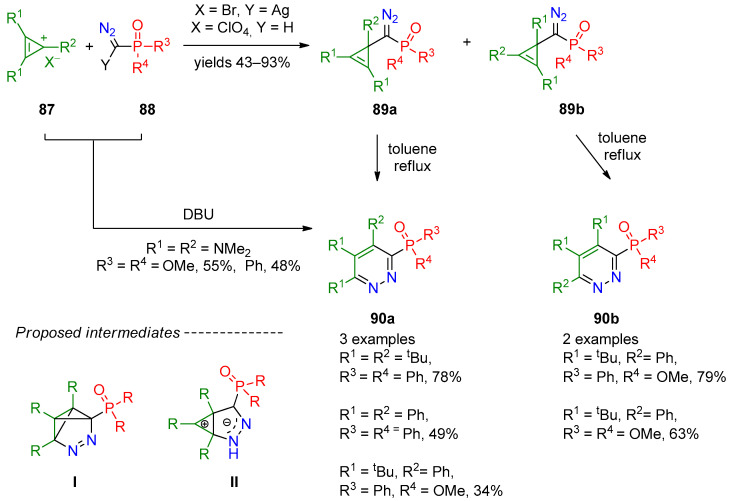
Reactions of cyclopropenylium salts with phosphorus-containing diazomethanes.

Regitz and co-workers [104,105,106,107] reported the only method for synthesis of POR_2_-containing pyridazines, the general character of which was demonstrated in relation to a representative series of compounds. The authors showed that (diazomethyl)cyclopropenes **89**, derived from cyclopropenylium salts **87** and phosphorus-containing diazomethanes **88**, undergo intramolecular isomerization to 3-P(O)R_2_-substituted pyridazines **90** (Scheme 22). When using secondary amino-substituted cyclopropenylium salts as substrates, the reaction can be performed in a one-pot fashion [107]. The method tolerates substrates containing phosphine oxide moieties and can be used to synthesize POR_2_-containg 4,5-diamino-substituted pyridazines in 16–68% yields. The authors suggested 3,4-diazabenzvalene **I,** product of the intramolecular 1,3-dipolar cycloaddition, or bicyclic zwitterions **II,** formed via intramolecular 1,5-dipolar cyclization, as two key intermediates in the isomerization reaction (Scheme 22). The results of the reactions with sterically hindered cyclopropenes inclined the authors to believe that the main reaction pathway involves intermediate **II**.

**Scheme 23:**
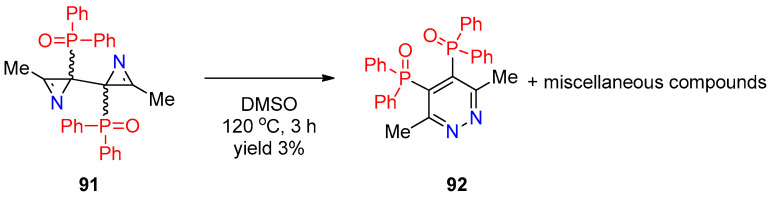
Rearrangement of phosphorus-containing bis-azirine.

A single example of thermal rearrangement of phosphorus-substituted bis-azirine **91** into 4,5-bis(phosphine oxide)pyridazine **92** was reported by Banert et al. [108] (Scheme 23). Formation of pyridazine may be explained by multistep diradical pathways, supported by DFT calculations. However, this is one of several reaction pathways, and the yield of the target product was as low as 3%. 

### 3.2. Intramolecular Cyclizations

**Scheme 24:**
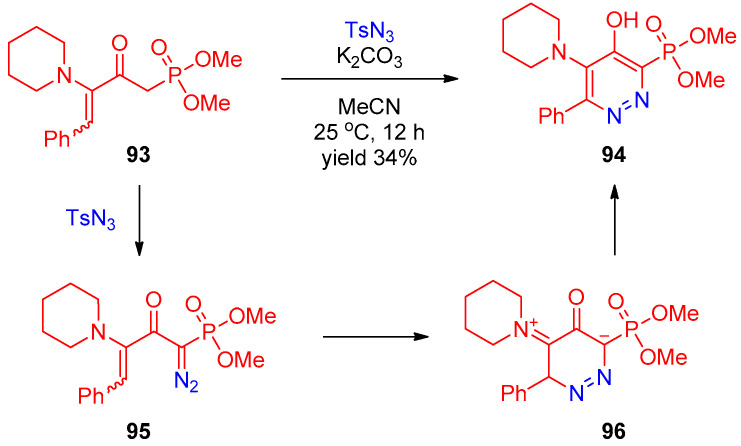
Cyclization of γ,δ-unsaturated α-diazo-β-keto phosphonate.

Doutheau and co-workers [109] described one example of cyclization of γ,δ-unsaturated α-diazo-β-keto phosphonate **95** to pyridazine-2-phosphonate **94** (Scheme 24). This compound is generated in situ via diazo transfer from γ,δ-unsaturated β-keto phosphonate **93** in the presence of tosyl azide and immediately undergoes intramolecular electrophilic cyclization involving a diazo moiety and double bond to form intermediate **96,** followed by rearrangement of the latter into final product.

**Scheme 25:**
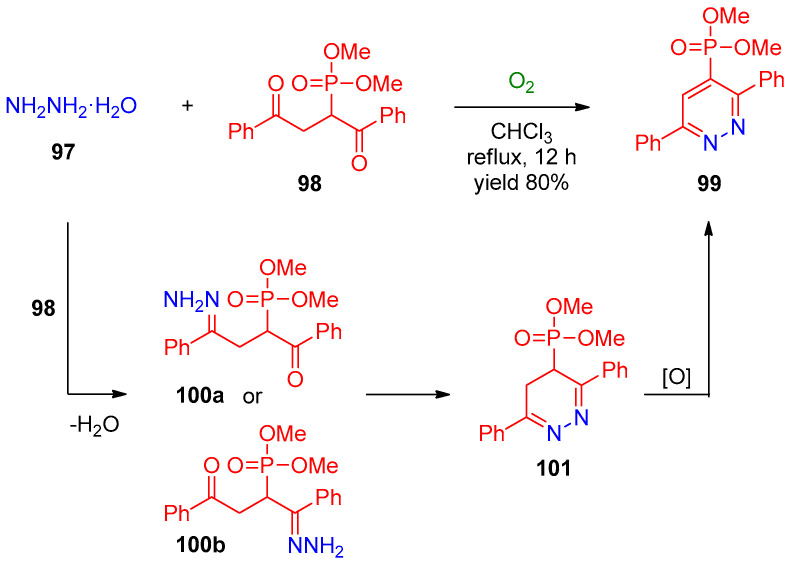
Synthesis of dimethyl 3,6-diphenylpyridazine-3-phosphonate.

Touil and Zantour [110] synthesized pyridazine-3-phosphonate **99** by reaction of phosphorylated 1,4-dicarbonyl compound **98** with hydrazine hydrate (**97**) under oxidative conditions (Scheme 25). The reaction presumably proceeds through intermediate hydrazones **100**, which undergo spontaneous intramolecular cyclization to 4,5-dihydropyridazine **101**. Oxidative aromatization of the latter with oxygen affords a product in high yield.

### 3.3. Phosphorylation of Pyridazines

**Scheme 26:**
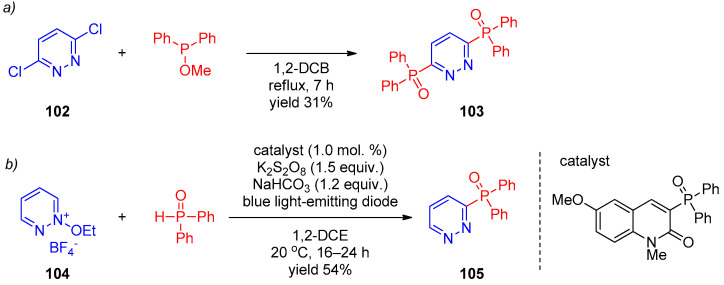
Phosphorylation of pyridazines: (**a**) Mrowca et al., 1980 [111]; (**b**) Kim et al., 2019 [79].

There are only two known examples of phosphorylation of pyridazines. Thus, the Michaelis–Arbuzov reaction of 3,6-dichloropyridazine (**102**) with methoxydiphenylphosphine was proposed as an approach to synthesis of 3,6-bis(diphenylphosphine oxide)pyridazine (**103**) in the patent by Mrowca [111] (Scheme 26, line a). An example of synthesis of 2-diphenyl(pyridazin-3-yl)phosphine oxide **105** from *N*-ethoxypyridazinium **104** and diphenylphosphine oxide via photocatalysis was presented by Kim et al. [79] (Scheme 26, line b). This process follows a radical mechanism, which is considered in more detail in Section 2.4.1 (Scheme 8). Therefore, the known phosphorylation reactions of pyridazines are limited to functionalization of the 3 and 6 positions of the heterocyclic core.

## 4. Pyrimidines

**Figure 3:**
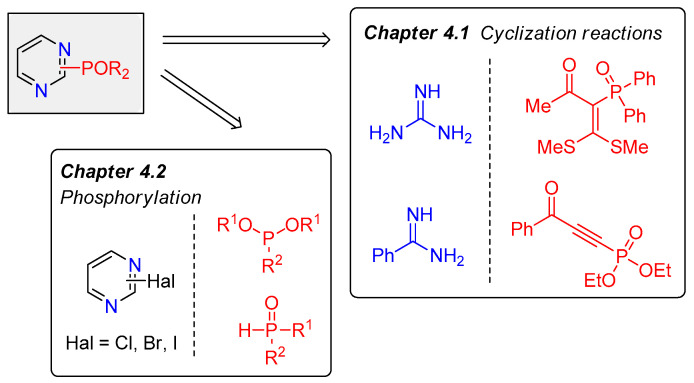
Approaches to synthesis of POR_2_-containing pyrimidines.

Phosphorus(V)-substituted pyrimidines were first synthesized by Kosolapoff and Roy [112] in 1961 by reaction of chloropyrimidines with NaP(O)(OEt)_2_. This method was also applied in many other studies [111,113]. Further, related compounds were prepared by reaction of bis-electrophiles with amidines [114,115,116,117,118], reaction of β-keto vinylphosphonates with amidines [119], reaction of metalated pyrimidines with P-Cl compounds [120], and palladium-catalyzed phosphorylation [45]. This section deals with recent examples of synthesis of phosphorus-containing pyrimidines, including the reactions of α,β-unsaturated β-phosphoryl carbonyl compounds with guanidine and phosphorylation of the pyrimidine core (Figure 3). 

### 4.1. Cyclizations Using Guanidine and Amidines

**Scheme 27:**
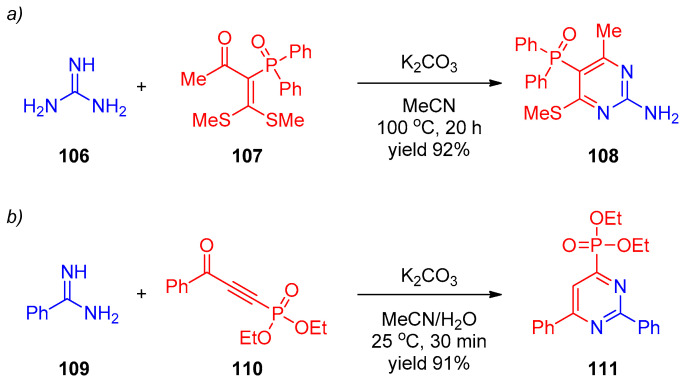
Cyclization reactions of guanidine (**a**) and amidine (**b**).

In early works, synthesis of pyrimidines was accomplished by heterocyclization of guanidine and amidines with phosphorus-containing bis-electrophiles, such as 3-(methylthio)acrylonitriles [114,118], 3-(dimethylamino)propenones [115,116], and acrylonitrile acetal [117]. Recently, Zhu et al. [121] reported one example of the reaction of phosphine oxide-substituted ketene dithioacetal **107** with guanidine (**106**) providing 5-phosphorus(V)-containing pyrimidine **108** (Scheme 27, line a). Liao et al. [68] described synthesis of pyrimidinyl-4-phosphonate **111** by a reaction between diethyl (3-phenyl-3-oxopropyn-1-yl)phosphonate **110** and benzamidine (**109**) (Scheme 27, line b).

In addition, it may be noted that, recently, three-component acidic condensation of β-keto phosphonates with aldehydes and urea (Biginelli reaction) was shown to be efficient for synthesis of 1,4-dihydro-2-oxopyrimidine-5-phosphonates [122,123,124]. In this regard, it can be assumed that, in the future, a similar approach using amidines can also be implemented to obtain pentavalent phosphorus-substituted pyrimidines.

### 4.2. Phosphorylation of Pyrimidines

Due to the significant limitations of synthetic approaches to structurally diverse phosphorus-substituted pyrimidines from phosphorus-containing precursors, phosphorylation of pyrimidines has gained great attention in recent years. Pioneering studies of nucleophilic and electrophilic phosphorylation of pyrimidines [111,112,113,120] were completed by the Michaelis–Arbuzov reaction, radical, and transition-metal-complex-catalyzed phosphorylation, which are discussed below.

#### 4.2.1. Michaelis–Arbuzov Reaction in Synthesis of Phosphorus-Substituted Pyrimidines 

**Scheme 28:**
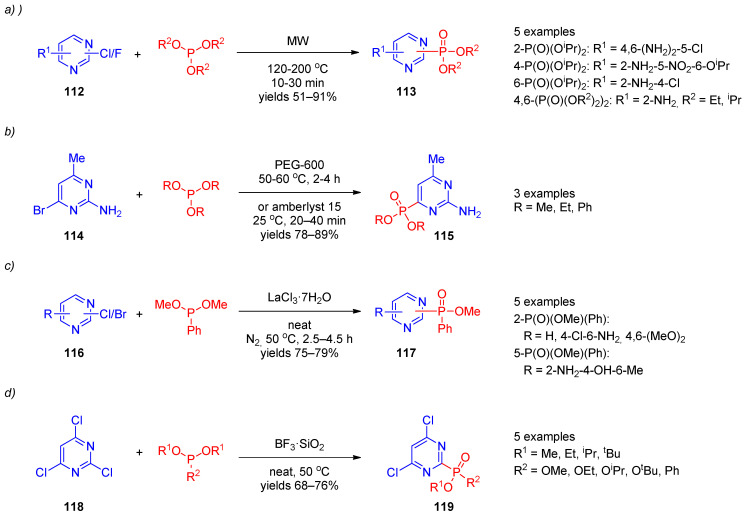
Michaelis–Arbuzov reaction in synthesis of pyrimidine phosphonates: (**a**) Jansa et al., 2012 [125]; (**b**) Kunda et al., 2011 [126]; Mohan Naidu et al., 2011 [127]; (**c**) Golla et al., 2014 [128]; (**d**) Varalakshmi et al., 2015 [129].

The Michaelis–Arbuzov reaction can probably be considered the most general method for synthesis of phosphorylpyrimidines known at the moment. Examples of use of the Michaelis–Arbuzov reaction in synthesis of pyrimidinylphosphonates (Scheme 28) were described in several studies [125,126,127,128,129]. Pyrimidines **113**, **115**, **117**, and **119** containing the phosphonate moiety at the 2, 4, 5, or 6 position were obtained by reaction of pyrimidinyl halides **112, 114, 116**, and **118**, respectively, with phosphites. Several procedures requiring either microwave activation (line a) or the presence of a catalyst, such as acidic ion-exchange resin (line b) or LaCl_3_ (line c), were developed and shown to be tolerant to electron-donating and electron-withdrawing substituents on the pyrimidine ring. The chemoselective Michaelis–Arbuzov reaction was accomplished by Varalakshmi et al. [129], who prepared 2,6-dichloropyrimidine-2-phosphonates by coupling of 2,4,6-trichloropyrimidine using silica-gel-supported BF_3_ as the catalyst (line d).

**Scheme 29:**
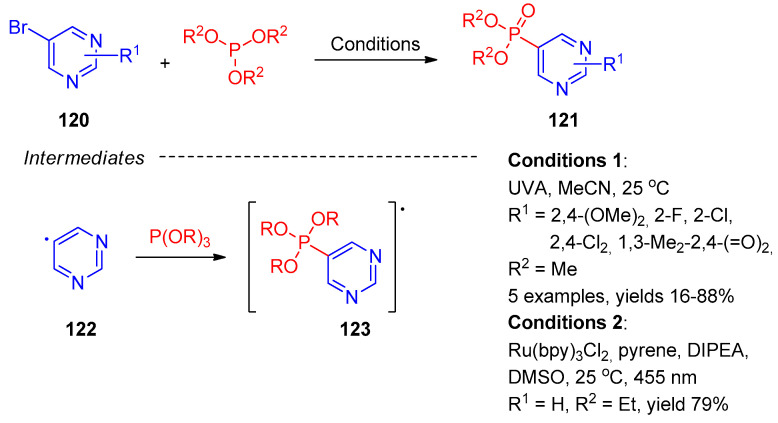
Photo-Arbuzov reaction in synthesis of pyrimidine-5-phosphonates.

A photochemical version of the Arbuzov reaction was shown to be efficient for synthesis of pyrimidine-5-phosphonates **121** from 5-bromopyrimidines **120** and trialkyl phosphites (Scheme 29) [130,131]. This radical process can be accomplished either directly under mild UV activation or using the Ru(bpy)_3_ pyrene dyad irradiated with blue light (455 nm). The plausible mechanism of this transformation involves formation of pyrimidinyl radical **122** either by action of an excited pyrene molecule or directly upon absorption of a light quantum. This radical attacks trialkyl phosphite, and the resulting radical **123** is decomposed to form the product accompanied by release of the alkyl radical. 

#### 4.2.2. Radical Phosphorylation of Pyrimidines

**Scheme 30:**
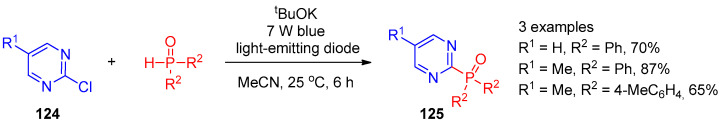
Photocatalytic phosphorylation of 2-chloropyrimidines.

Radical functionalization of pyrimidines with phosphine oxides and phosphonates is poorly known. Only the photocatalytic radical reaction of 2-chloropyrimidines **124** with secondary phosphine oxides giving products **125** (Scheme 30) was recently described by Yuan et al. [73] The mechanism of this transformation is similar to that for chloropyridines and is considered in Section 2.4.1 (Scheme 6). As a distant example of the radical phosphorylation of pyrimidines, let us mention the work by Zhang et al. [132], who accomplished phosphorylation of pyrimidin-4-ones with dimethyl phosphite in the presence of Mn(OAc)_3_.

#### 4.2.3. Transition-Metal-Catalyzed Phosphorylation of Pyrimidines

**Scheme 31:**
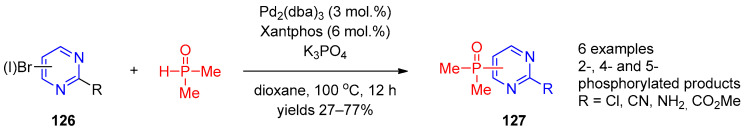
Pd_2_(dba)_3_-catalyzed cross-coupling of halopyrimidines with dimethylphosphine oxide.

Since the first work on palladium-catalyzed phosphorylation of pyrimidines published in 2008 by Belabassi et al. [45], transition-metal-catalyzed cross-coupling of halopyrimidines with secondary phosphine oxides and phosphonates proved to be an efficient route to phosphorylpyrimidines. Mykhailiuk and co-workers [91] published a procedure for Pd(II)-catalyzed coupling of halo(Br/I)pyrimidines **126** with dimethylphosphine oxide, showing remarkable generality (Scheme 31). Synthesis of 2-, 4-, and 6-phosphine-oxide-substituted pyrimidines **127** bearing electron-donating and electron-withdrawing substituents on the heterocyclic moiety was accomplished using Pd_2_(dba)_3_ in the presence of the Xantphos ligand.

**Scheme 32:**
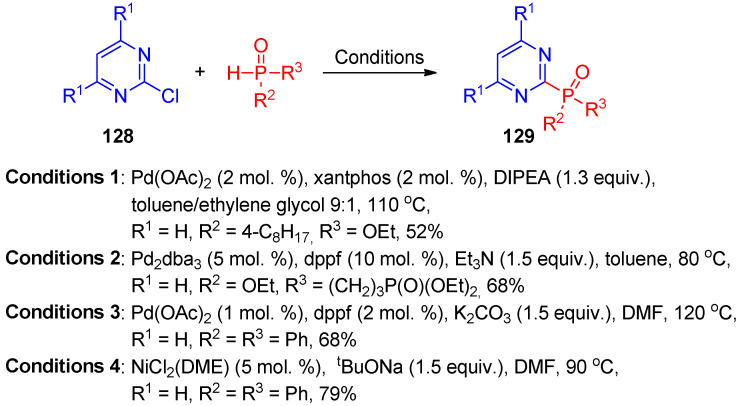
Transition-metal-catalyzed cross-coupling of 2-chloropyrimidines with phosphine oxides.

Individual examples of metal-complex-catalyzed coupling of 2-chloropyrimidines **128** with phosphine oxides were published in studies by Montchamp, Yang, Montel, Zakirova, and their co-workers (Scheme 32) [133,134,135,136]. Catalysis was accomplished using Pd(II), Pd(0), or Ni(II) salts, providing 2-phosphorylated pyrimidines **129** in good yield. 

**Scheme 33:**
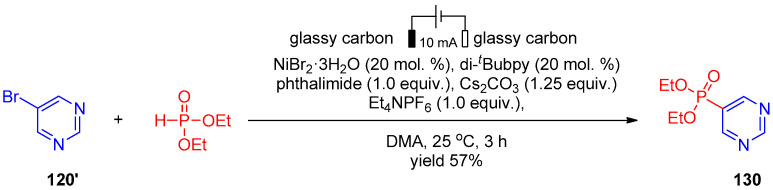
NiBr_2_-catalyzed electrochemical synthesis of 5-phosphorylpyrimidine.

Bai et al. [137] reported one example of electrochemical phosphorylation of 5-bromopyrimidine (**120′**) with diethyl phosphonate in the presence of NiBr_2_ (Scheme 33), giving diethylpyrimidine-5-phosphonate **130**. The authors suggested that the reaction proceeds via a radical mechanism accompanied by anodic generation of a phosphorus-centered radical.

## 5. Pyrazines

**Figure 4:**
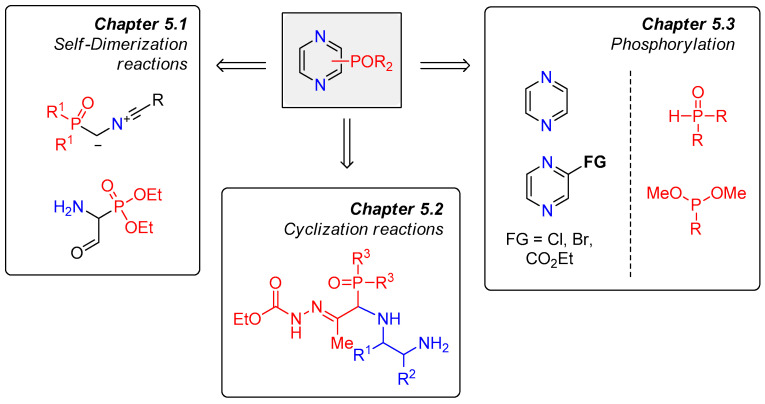
Approaches to synthesis of POR_2_-containing pyrazines.

Phosphorus(V)-containing pyrazines are poorly studied six-membered *N*-heterocycles. Synthesis of phosphorylpyrazines was described or mentioned in just over a dozen publications, and, what is more important, most of them are fragmentary in scope. The group of Palacios in the 2000s [138,139,140]. made the most significant contribution to the development of original synthetic approaches to POR_2_-substituted pyrazines. The authors proposed self-dimerization of nitrile ylides and 4-dimethylamino-3-phosphoryl-2-azadienes and the formal [4+2]-cycloaddition of 1,2-diaza-1,3-butadienes with 1,2-diamines, which are considered below along with works on phosphorylation of the pyrazine ring (Figure 4). 

### 5.1. Dimerization Reactions

**Scheme 34:**
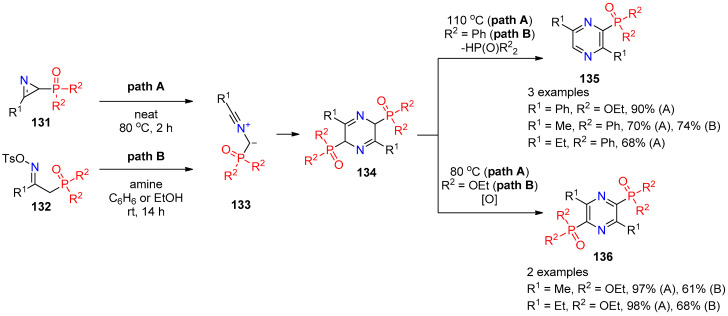
Synthesis of phosphorus-containing pyrazines via dimerization of nitrile ylides.

Palacios et al. [138] described synthesis of phosphorus-substituted pyrazines via dimerization of nitrile ylides **133** (Scheme 34), which can be generated in situ from POR_2_-bearing *2H*-azirines **131** (pathway A) or tosylated 2-hydroxyiminophosphonates **132** (pathway B). According to the proposed mechanism, intermediate **133** undergoes self-dimerization to dihydropyrazine **134** followed by either oxidation of the latter compound or elimination of secondary phosphine oxide. An interesting feature of this transformation is that it allows targeted synthesis of either mono- or bisphosphorus(V)-substituted pyrazines **135** and **136**. Thus, synthesis via pathway A at 80 °C affords products **136**, whereas heating to 110 °C yields products **135**. The outcome of the reaction via pathway B is determined by substituents on the phosphorus(V) moiety. Thus, presence of a phenyl group results in formation of the monophosphorylated product, whereas the bisphosphorylated product is generated in the presence of the ethoxy group. 

**Scheme 35:**
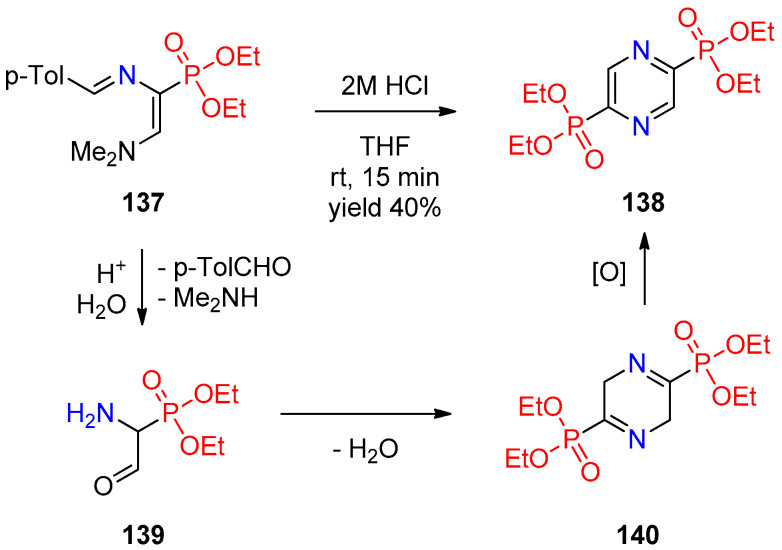
Dimerization of 4-dimethylamino-3-phosphoryl-2-azadiene.

In another study, the same group [139] demonstrated that bis-2,5-phosphorylpyrazine **138** can be obtained via dimerization of aminoaldehyde **139** derived from 4-dimethylamino-3-phosphoryl-2-azadiene **137** via acid hydrolysis of both enamine and imine groups (Scheme 35). The reaction was proposed to occur through formation of dihydropyrazine **140**, the oxidation of which in the reaction medium provides the final pyrazine.

### 5.2. Formal [4+2]-Cycloaddition

**Scheme 36:**
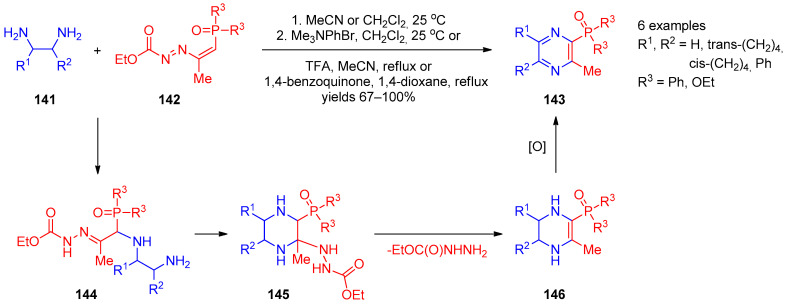
Synthesis of 2-POR_2_-substituted pyrazines from 1,2-diaza-1,3-butadienes and 1,2-diamines.

In the subsequent study, Palacios and co-workers [140] used the reaction of phosphorus-containing 1,2-diaza-1,3-butadienes **142** with 1,2-diamines **141** as access to polysubstituted POR_2_-modified pyrazines **143** (Scheme 36). This formal [4+2]-cycloaddition is a general method for synthesis of alkyl- and arylpyrazines bearing phosphine oxides and phosphonates substituents. The plausible mechanism of this reaction involves the following steps: the Michael addition of diamine **141** to diazadiene system **142,** giving adduct **144**, intramolecular attack of the second amino group on the C=N double bond of hydrazone, and elimination of ethyl hydrazinecarboxylate from the resulting piperazine **145** followed by oxidation of 1,2,3,4-tetrahydropyrazine **146** to pyrazine.

### 5.3. Phosphorylation of Pyrazines

Synthesis of 2-phosphorus(V)-substituted pyrazine based on phosphorylation was first described by Seggio et al. [141] in 2007. They reported an efficient two-step procedure for preparation of (diphenylphosphino)pyrazine oxide based on deprotonation of pyrazine with a mixture of ZnCl_2_‚ TMEDA, and LiTMP followed by treatment of the resulting lithium complex of di(pyrazin-2-yl)zinc with chlorodiphenylphosphine. Most of the later studies are scattered and describe single examples of phosphorylation of the pyridazine core via the Michaelis–Arbuzov reaction, radical, and transition-metal-catalyzed phosphorylation reactions, which are discussed below.

#### 5.3.1. Michaelis–Arbuzov Reaction in the Synthesis of Phosphorus-Substituted Pyrazines

**Scheme 37:**
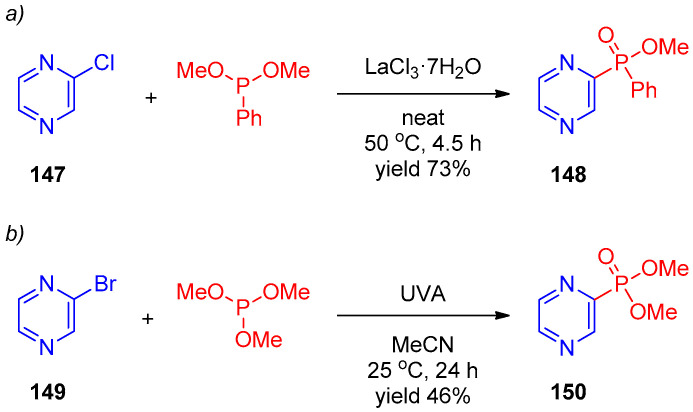
Synthesis of 2-phosphorylpyrazine by the Arbuzov reaction: (**a**) Golla et al., 2014 [128]; (**b**) Goddard et al., 2016 [131].

Despite more than a century of history, the Michaelis–Arbuzov reaction was relatively recently applied for phosphorylation of pyridazines. Thus, Golla et al. [128] synthesized 2-phosphorylpyrazine **148** from 2-chloropyrazine (**147**) and dimethyl phenylphosphonite using LaCl_3_•7H_2_O as the catalyst under neat conditions (Scheme 37, line a). Goddard and co-workers [131] reported an example of using the photo-Arbuzov reaction to prepare dimethyl pyrazine-2-phosphonate **150** from 2-bromopyrazine (**149**) and trimethyl phosphite under mild UVA irradiation (Scheme 37, line b). 

#### 5.3.2. Radical Phosphorylation of Pyrazines

**Scheme 38:**
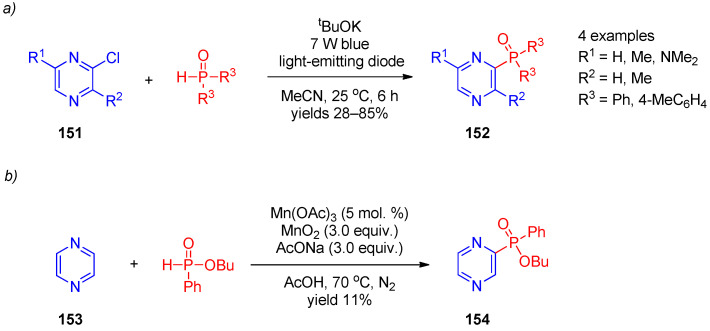
Synthesis of 2-POR_2_-substituted pyrazines by radical phosphorylation: (**a**) Yuan et al., 2018 [73]; (**b**) Berger and Montchamp, 2019 [142].

Recently, Yuan et al. [73] described three examples of photocatalytic radical phosphorylation of 2-chloropyrazines **151** with secondary phosphine oxides, giving 2-substituted pyrazines **152** (Scheme 38, line a). The reaction occurred in the presence of ^t^BuOK under mild conditions using irradiation with a blue-light-emitting diode. The mechanism of transformation is similar to that discussed for pyridines in Chapter 2.4.1 (Scheme 6). Further, Berger and Montchamp [142] reported synthesis of single 2-phosphorylated pyrazine **154** by reaction of pyridazine (**153**) with phosphonite in the presence of MnO_2_ and Mn(OAc)_3_ (Scheme 38, line b). The authors suggested that the reaction proceeds through formation of a phosphorus-centered radical that adds to pyrazine.

#### 5.3.3. Transition-Metal-Catalyzed Phosphorylation of Pyrazines

**Scheme 39:**
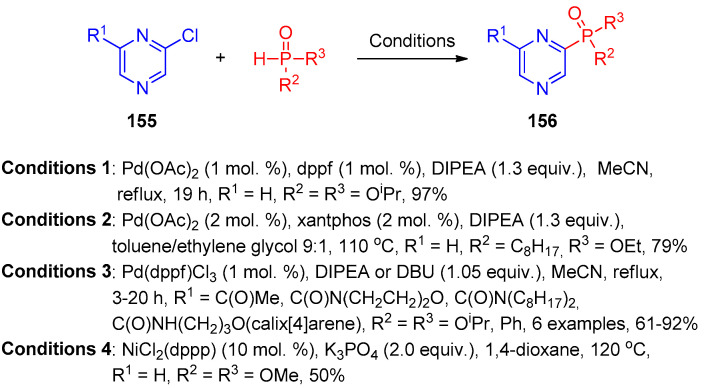
Synthesis of 2-phosphorylpyrazines under transition metal catalysis.

It was shown that 2-chloropyrimidines **155** have good synthetic potential as precursors for synthesis of 2-phosphorus-substituted pyrazines **156** under conditions of metal-catalyzed phosphorylation (Scheme 39). The Pd(II)-catalyzed phosphorylation of chloropyrazine was described in studies by Belabassi, Deal, Nikishin, and their co-workers [40,45,133,143] (conditions 1–3). The generality of the approach with respect to phosphine oxides and phosphonates was demonstrated using Pd(dppf)Cl_3_ as the catalyst (condition 3). Note that Zhao et al. [144] performed this transformation in the presence of NiCl_2_(dppp) complexes as the catalyst (condition 4). Further, Yamaguchi and co-workers [100] described one example of nickel(II)-acetate-catalyzed phosphorylation of phenyl pyrazine-2-carboxylate with diphenylphosphine oxide accompanied by decarbonylation.

## 6. Conclusions

In conclusion, in the past decade, significant progress was made in synthesis of phosphorus-substituted six-membered aromatic heterocycles. Numerous interesting synthetic approaches were developed based on construction of a heterocyclic core using phosphoryl-containing reagents, mainly formal cycloaddition and intra(inter)molecular cyclization/isomerization, using the Michael reaction, carbenoid intermediates, and classical nucleophilic–electrophilic interactions. Their apparent advantages are structurally diverse products and high tolerance to functional groups. Further, phosphorylation represents the most general route to phosphorus-substituted pyridines, pyridazines, pyrimidines, and pyrazines. Halogen-substituent in six-membered *N*-heterocycles has proven to be a universal leaving group providing phosphorylation, and some functional groups (OH, OTs, B(OH)_2_, CO_2_Ph, N=NSO_2_Me, etc.) were also efficient in this process. However, the necessity of finding conditions for each particular type of heterocycles, severe reaction conditions (high temperature, presence of strong bases and acids), and high cost of reagent/catalysts significantly limit practical application of this approach.

Evidently, development of synthetic methods based on phosphorylation of heterocycles and heterocyclization involving phosphorus-substituted reagents is of fundamental research interest. In point of fact, pyridines have received a great deal of attention among monocyclic six-membered *N*-heterocycles in the past decade, resulting in development of a number of approaches with good substrate scope and efficiency. Most of the methods proposed for pyridazines, pyrimidines, and pyrazines are fragmented and sporadic, while phosphorus-substituted triazines and tetrazines are not described. In this regard, further development of this research area is associated with the following issues: *design of new versatile multifunctional reagents for various chemical transformations* (detailed investigation of such new reagents would provide the basis for a new area in synthesis of nitrogen-containing heterocyclic compounds with different combinations of heteroatoms and also for preparation of new complex heterocyclic systems and assemblies); *transition-metal-catalyzed C–H phosphorylation of six-memebered N-heterocycles* (transition-metal-catalyzed CH phosphorylation is a straightforward and attractive approach to construct C–P bonds; however, it is difficult to execute this approach in practice because of the strong propensity of phosphorus reagents to induce catalyst poisoning through coordination); *development of methods for synthesis of phosphorus-substituted six-memebered N-heterocycles with more than two nitrogen atoms in the ringe* (no representatives of such compounds have been described to date). Since there are a few examples of synthesis of certain phosphorus(V)-substituted six-membered heterocycles while synthetic approaches to phosphorylated derivatives of a wide range of particular *N*-heterocycles are still absent, further research in this field is a long-term challenge.

## Data Availability

Not applicable.

## Abbreviations

N-methyl-D-aspartate (NMDA); dibenzylideneacetone (dba); 1,8-diazabicyclo [5.4.0]undec-7-ene (DBU); density functional theory (DFT); 1,1′-bis(diphenylphosphino)ferrocene (dppf); 1,3-bis(diphenylphosphino)propane (dppp); 4,4’-dipyridyl (dpy); lithium tetramethylpiperidide (LiTMP); tetramethylethylenediamine (TMEDA); ultraviolet radiation (UVA); 4,5-bis(diphenylphosphino)-9,9-dimethylxanthene (Xantphos).
